# Increasing Collaborative Discussion in Case-Based Learning Improves Student Engagement and Knowledge Acquisition

**DOI:** 10.1007/s40670-022-01614-w

**Published:** 2022-09-05

**Authors:** Nana Sartania, Sharon Sneddon, James G. Boyle, Emily McQuarrie, Harry P. de Koning

**Affiliations:** 1 Undergraduate Medical School, School of Medicine, University of Glasgow, Glasgow, UK; 2 Institute of Infection, Immunity and Inflammation, University of Glasgow, Glasgow, UK

**Keywords:** Medical education, Case-based learning, Collaborative learning, Small group teaching

## Abstract

**Background:**

In the transition from academic to clinical learning, the development of clinical reasoning skills and teamwork is essential, but not easily achieved by didactic teaching only. Case-based learning (CBL) was designed to stimulate discussions of genuine clinical cases and diagnoses but in our initial format (CBL’10) remained predominantly tutor-driven rather than student-directed. However, interactive teaching methods stimulate deep learning and consolidate taught material, and we therefore introduced a more collaborative CBL (cCBL), featuring a structured format with discussions in small breakout groups. This aimed to increase student participation and improve learning outcomes.

**Method:**

A survey with open and closed questions was distributed among 149 students and 36 tutors that had participated in sessions of both CBL formats. A statistical analysis compared exam scores of topics taught via CBL’10 and cCBL.

**Results:**

Students and tutors both evaluated the switch to cCBL positively, reporting that it increased student participation and enhanced consolidation and integration of the wider subject area. They also reported that the cCBL sessions increased constructive discussion and stimulated deep learning. Moreover, tutors found the more structured cCBL sessions easier to facilitate. Analysis of exam results showed that summative assessment scores of subjects switched to cCBL significantly increased compared to previous years, whereas scores of subjects that remained taught as CBL’10 did not change.

**Conclusions:**

Compared to our initial, tutor-led CBL format, cCBL resulted in improved educational outcomes, leading to increased participation, confidence, discussion and higher exam scores.

## Introduction

Teaching methods in medical education that involve students in discussion and interaction continue to evolve with a focus on promoting collaborative and active learning. Many medical schools have introduced clinical teaching early in the curriculum in an attempt to integrate basic and clinical sciences [1]. The use of clinical cases to aid teaching has been documented for over a century, with the first use of case-based learning (CBL) in 1912 to teach pathology at the University of Edinburgh [2]. There is no set definition for CBL currently. Thistlethwaite et al. [3] define the goal of CBL ‘is to prepare students for clinical practice through the use of authentic clinical cases. It links theory to practice through the application of knowledge to the cases, using inquiry-based learning methods’, and it fits in the continuum between structured and guided learning. As such, ‘CBL’ comes in many different formats, some more didactic, some more participatory, but the pros and cons of the different approaches have rarely been systematically evaluated.

Clinical Reasoning is one of the key skills to be taught before the transition into the clinic. Diagnostic error rates continue to be high [4] and reflect deficits in both knowledge and reasoning skills. Training in clinical reasoning enhances the students’ ability to transfer declarative knowledge to clinical problems in preparation for working in clinical teams, and a format of transition-stage teaching that addresses both deficits in tandem would be highly beneficial. It has been reported that some forms of CBL learning compare favourably to didactic teaching [5–7] because it is a participatory method [8] that leads to improved motivation [9] and the development of reflective thinking [10]. Preparing students to think like clinicians before they commence clinical attachments affords opportunities for vertical and horizontal integration of the curriculum and fosters learning for competence [11].

CBL was introduced at the University of Glasgow, in its initial format, in 2010 (CBL’10) and incorporated in ‘Phase 3', a 15-week-long period of transition into full-time clinical teaching with a focus on pathophysiology. McLean [7] reviewed several published definitions of CBL and summarized that CBL requires the presentation of a clinical case followed by an ‘enquiry’ on the part of the learner and provision of further information by a tutor who guides the discussion towards meeting the learning objectives—the idea we tried to replicate in CBL’10. However, our in-house evaluation found that student participation was uneven, and the sessions often became too didactic, contravening the idea of CBLs being student-centred. As a form of adult learning, Mayo [12] describes CBL in terms of the socio-constructivist model, with the students themselves constructing the new knowledge and insights and the tutor functioning merely as a guide.

**Fig. 1:**
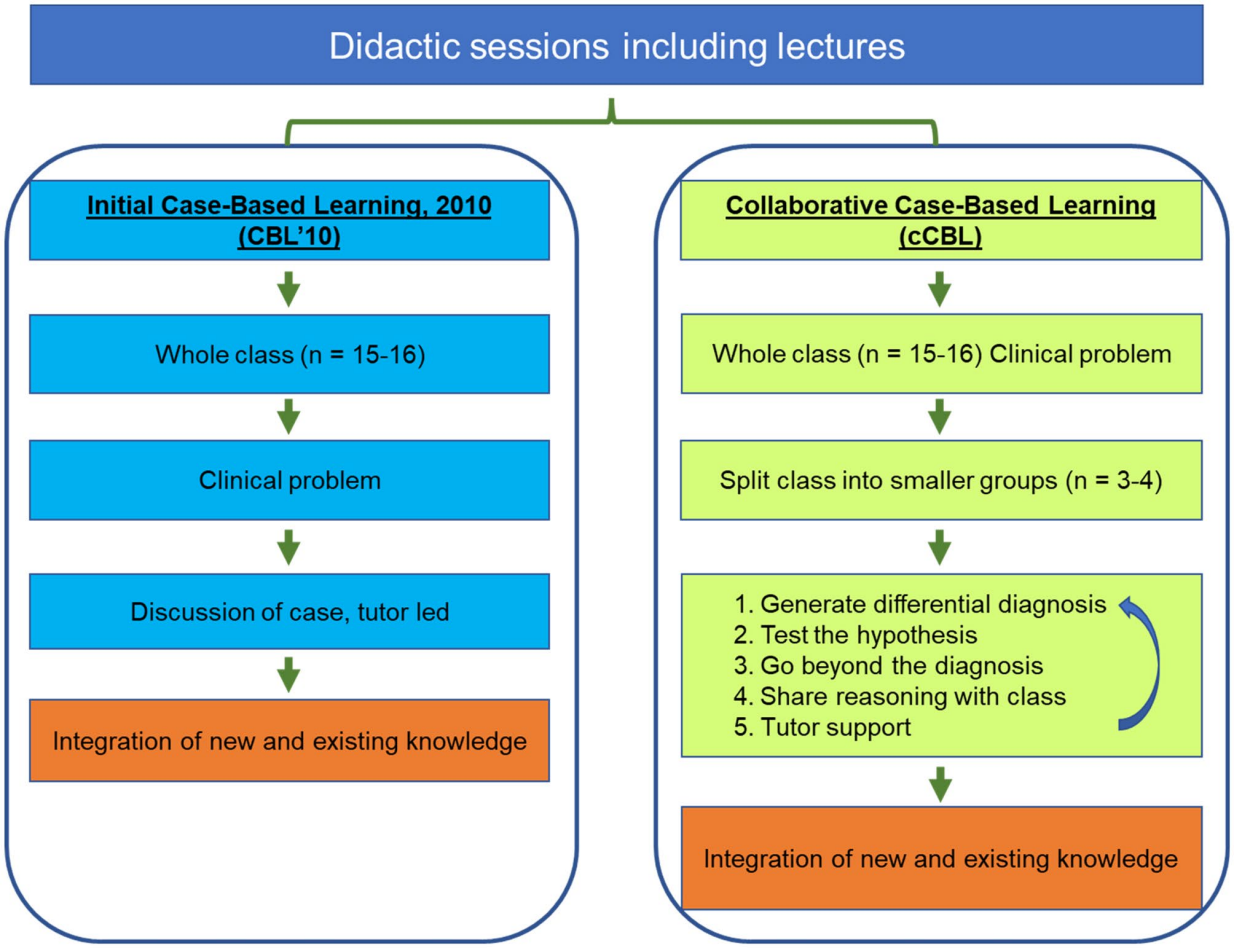
Comparison of the CBL’10 and collaborative CBL formats. An essential feature of our cCBL is the iterative nature of the small group discussion with the small groups reporting back to class, leading to next-level case information being released by the tutor followed by additional round(s) of small group discussions

The recent work by Schwartzstein and colleagues [13] suggested that collaborative case-based learning (cCBL), a modification of CBL, brings additional benefits to students over more didactic forms of CBL in terms of enjoyment and working collectively in teams. Addition of classroom discussions to case-based learning encourages the students to make inferences and conclusions from the presented data [14, 15]. cCBL is described as ‘team-based’ and as incorporating elements of both PBL and CBL [13]. In PBL, however, a problem is presented as the means of obtaining basic scientific knowledge, whereas CBL is typically supported by prior didactic teaching to assure the class has the required knowledge base to discuss the clinical case; they exercise logical diagnostic problem-solving that combines comprehension, critical thinking and problem-solving skills, engendering deep cognitive learning [16]. cCBL places more emphasis on student-based discussions of focused but open-ended questions in small groups, before reaching a consensus in a larger group, and requires students to iteratively generate their own hypotheses from real-life clinical observations and data (Fig. 1), incorporating elements of various small group teaching modalities [13]. The collaborative format integrates cognitive and social learning modes [12] and makes the material appear more relevant [17], particularly for students with below average academic achievement and/or hesitant to participate in discussions in large groups. A key benefit of cCBL is the increased interactivity between the students in small groups to ensure student-centred active learning and reasoning. The sessions require the students to integrate different types of information, including clinical and social data and ethical considerations, while the training in evidence-based deduction stimulates the procedural learning essential for clinical reasoning [18, 19]. The CBL’10 and cCBL formats are compared in Fig. 1.

We here present a pilot study where we re-designed some of our CBL’10 scenarios to fit the cCBL format in which we reinforced the discussion element, using breakout groups for intensive small group discussions, while keeping the other modules in the CBL’10 format. The collaborative approach aimed to encourage students to practice clinical reasoning and decision-making by providing iterative experiences of analysing and problem-solving complex cases. This models genuine care situations where clinicians are required to integrate a variety of topics, including ethical issues, prevention and epidemiology. Students are given relevant case information to discuss, from which they form hypotheses. They report back to the full class, upon which further information is released (e.g. ‘investigative outcomes’) followed by subsequent discussion in the breakout groups; this process is repeated several times (Fig. 1).

In 2015, the National Academy of Medicine urgently highlighted the need to improve diagnostic error rates, which continued to be too high [20]. The change to cCBL aimed to improve inductive clinical reasoning skills and joint decision-making in small teams. Moreover, this format encourages the ‘explicit integration’ of clinical reasoning in the undergraduate years of the medical curriculum, as encouraged in a recent consensus statement by the UK Clinical Reasoning in Medical Education (CReME) group in order to foster effective clinical reasoning in teams [21]. Importantly, we believe that the collaborative format also highlights the importance of a truly patient-centred care to participating students, which may not be as evident in CBL’10.

While it is expected that cCBL encourages a higher level of participation and should result in better knowledge as well as better reasoning and decision making in clinical practice, it is not easy to summatively assess clinical reasoning in an undergraduate setting. Indeed, a systematic review of the use of CBL, drawing on worldwide practice, shows that the three top methods of evaluation of a CBL learning session were survey (36%), test (17%) and test plus survey (16%) [7]. OSCE was used less frequently—only 9% of the studies reviewed reported using a practical exam like OSCE [7] or prescribing [22] as an outcome for evaluating the effectiveness of CBL. Here, we share a mixed-methods evaluation of our implementation, consisting of both a survey focusing on issues of participation and motivation and on summative assessments to gauge the effects on integrative knowledge acquisition in subjects taught by either CBL format.

## Methods

This pilot study focuses on the comparison of CBL’10 and cCBL as used in Phase 3 of the Glasgow Undergraduate Medical School curriculum. For this study, we have converted three CBL’10 scenarios to cCBL, while other sessions continued unchanged as CBL’10. Evaluations by students and tutors of these sessions are presented, alongside a side-by-side comparison of the exam performance for topics taught by the two CBL formats. All tutors are trained by faculty and had experience of teaching both versions of CBLs and were as such, well acquainted with both methods when asked to compare the two. Similarly, all students surveyed had participated in both formats.

After presenting the initial case information to the class of 16 students, the group was divided into sub-groups of 3–4 students each. The cCBL included pre-class readiness assessment and in class activities in which students were asked to work on specific tasks individually (discuss differentials; propose investigations; suggest treatment and management), debate their answers in groups of 3–4 and record these on ‘post-it’ notes presented to the full class as part of the discussion to reach a consensus in the larger groups of 16 [13].

Students (*n* = 270) took part in a survey that gathered quantitative and qualitative data from responding year 3 students (Table 1; *n* = 149; 55%) and clinical tutors (Table 2; *n* = 36). The questionnaire addressed the participants’ perception of the effectiveness of the cCBL sessions. The survey was designed by NS and SS. The questions were based on the annual course evaluation forms and included core statutory questions used for teaching appraisal as well as bespoke ones specific to cCBLs. The survey has high face validity as the authors have extensive experience of questionnaire design, are experts in CBL and are highly experienced in curriculum design, development and evaluation.

**Table 1:** Descriptive statistics (student responses, *n* = 149). Questions were asked on 5-point Likert scale: 1, strongly disagree (SD); 5, strongly agree (SA). SA and A (agree) were grouped together. Similarly, SD and D (disagree) were combined

	SA/agree	Neutral	SD/disagree	Total	**%** of total SA/A
I noticed a difference in the way the CBL session ran today	118	23	8	149	**79**
The CBL: session created an environment that encouraged group discussion to assist learning	134	7	8	149	**90**
The CBL session felt more interactive	122	20	7	149	**82**
The tutor interacted with students in the group, directly answering questions, and leading their learning to achieve the stated learning objectives	142	6	1	149	**95**
The breakout sessions were useful in discussing topics with my peers	111	25	12	148	**75**
This CBL session helped me understand the topic better	136	9	4	149	**91**
There was enough time to discuss all the information in the case	133	4	12	149	**89**

**Table 2:** Descriptive statistics (tutor responses, *n* = 36). Tutors' reflection on cCBL/CBL’10

	SA/agree	Neutral	SD/disagree	Total	% of total SA/A
The CBL sessions felt more interactive	27	8	1	36	**75**
The session created an environment that encouraged group discussion	33	3	0	36	**92**
The tutor material provided helped me run the session efficiently	27	5	4	36	**75**
I felt able to lead the students in their learning to achieve the stated learning objectives	34	2	0	36	**94**
There was enough time to discuss all the information in the case	31	3	2	36	**86**
The breakout sessions were useful to get students to discuss the case with their peers	33	1	2	36	**92**

The paper-based surveys were distributed and collected by the year administrator immediately after the cCBL teaching sessions and contained open and closed questions (5-point Likert scale: 1, strongly disagree; and 5, strongly agree) on the experiences of the group work, peer-to-peer interaction and the intended learning outcomes (ILOs) coverage throughout these sessions. Open questions in the survey were categorized into sub-themes through an inductive process, and the dominant thematic categories were agreed upon and analysed. Quotes were selected in relation to the whole dataset. Initial subthemes were refined through successive returns to the data, from which additional quotes were used.

We applied interpretive analysis to develop an argument based on the categories identified, in order to explain how the use of cCBLs has helped students to overcome team-learning difficulties using collaborative inductive reasoning. This nested mixed method [23, 24] provides a stronger basis of causal inference by combining quantitative analysis of the large open question dataset with an in-depth investigation of the student and tutor responses embedded in the survey.

The second outcome measure was a comparative analysis of the end-of-year exam performance between two independent cohorts that were assessed on the topics covered in cCBL versus the same topics taught via CBL’10 in previous years. The three cCBL-taught topics assessed in 2019/2020 and 2020/2021 were myocardial infarction, chronic kidney disease and diabetes; the outcomes were compared to the results of 2015/2016 and 2018/2019, when students were assessed on the same topics, then taught via CBL’10. Student knowledge was assessed in summative exams using both multiple choice questions (MCQ) and modified essay questions (MEQ), with a similar weighting for each component. Exam questions were developed and standard set by subject experts involved in designing and delivering the curriculum and quality assured to ensure validity and reliability. Exam questions were subject to internal and external scrutiny. Questions were blueprinted to intended learning outcomes (ILOs) to ensure content validity and that the type of testing used (MEQs) is appropriate to test relevant knowledge in terms of diagnosis, investigation and management in a written format (Downing et al., 2003) [25]. MEQs are often used to assess higher order abilities and abstract knowledge according to Bloom’s taxonomy, for which they are preferred over MCQs [26]. For example, structured questions are mapped to intended learning outcomes such as the ability to ‘differentiate’, which requires analysing and conceptualizing knowledge [27]. Examinations were standard set using a modified Angoff method whereby the panel judges (subject experts) discuss each question and arbitrate the expected answers from students [28]. Information regarding internal consistency of the exams was determined by calculating Cronbach’s alpha [29]. Data are presented as mean exam score ± SE. An unpaired, two-tailed *t* test was used to establish whether the difference in knowledge gain of the topics taught by the two methods is indeed attributable to the new teaching method. *p* < *0.01* was considered statistically significant.

## Results

### The Practice of CBL at the University of Glasgow

The three re-designed collaborative CBLs introduced in 2019/20 were compared to the remaining unchanged CBL’10 s. The students took part in 90-min CBL sessions twice a week and all participated equally in both types of CBL sessions and all other aspects of the course. The key differences between the two types of CBL are shown in Table 3.

**Table 3:** Differences between the CBL’10 and cCBL formats

	**CBL’10**	**cCBL**
**Goal**	Learn about the disease and the complexity of care; multifaceted approach	Students will formulate their own hypothesis, analyze the case and suggest investigations and care options; Collaborative learning; Clinical reasoning
**Focus**	Knowledge acquisition using clinical cases as teaching tools	Acquisition of knowledge as well as clinical understanding. Understanding how the healthcare system works cooperatively
**Group size**	12–14	12–16 with subdivisions into groups of 3–4
**Role of learner**	Expected to ask questions; willingness to discuss the case; recall of previous knowledge	Active participation necessary; application of newly acquired knowledge from lectures; collaborative approach, iteratively developing and adjusting an evidence-based hypothesis
**Role of tutor/faculty**	Guide the discussions, give own perspective, share knowledge/ expertise; ensure ILOs are covered; act as a resource	Present the case; guide task-related discussions; provide case-related information; ensure ILOs are met; correct any misconceptions by learners; sum up the case
**Learning objectives**	Pre-set learning objectives	Learning objectives may develop in-session when new dimensions are added as a result of discussions
**Outcomes**	Learning about the case; integrate with the knowledge acquired in lectures prior to CBL	Training in reasoning and deductive processes; familiarity with the multi-faceted aspects of clinical decision making

In our CBL’10 s, tutors led a more didactic session with the occasional discussions designed to allow the students to contextualize the knowledge gained from earlier lectures while still building on the clinical and problem-solving aspects of the medical curriculum.

In cCBLs, students were expected to follow the structured script and asked to formulate hypotheses. This required them to apply the knowledge gained from the supporting lectures and other teaching formats to various clinical presentations in three steps. First, they needed to generate a differential diagnosis and commit to a most likely diagnosis. The next step was to test the hypothesis and see what additional tests they required in order to confirm or reject the initial diagnosis. Finally, students are asked to go beyond the diagnosis and explain their therapeutic goal, as well as consider the factors that might influence the patient’s response to therapy. Students feed their thoughts back to the main group using post-it notes.

Groups of 16 were further subdivided into smaller groups of 3–4 students each, which were given specific tasks (e.g. generating differential diagnosis; proposing investigations and/or possible treatment/management). Through interactions in the small break-out groups, students were able to integrate a variety of topics, including prevention, ethical issues and epidemiology or health systems issues and generate discussions necessary for confirming or refuting the initial diagnosis. The advantage of smaller groups was the opportunity to practice analytical reasoning skills in a safe environment. In terms of group size in shared reasoning, Edelbring et al. [30] found the dyad peer setting works best, although there was a concern that any knowledge asymmetry in the peer group may negatively impact on the learning experience.

### Survey of Opinion of Collaborative Versus Traditional CBL Sessions

Qualitative analysis identified two overarching themes: enhanced engagement and gain of knowledge.

1. Enhanced engagement

Students and tutors both found that cCBLs increased student engagement and motivation for self-directed learning as a result of the more interactive nature of these sessions. Ninety percent of students and 92% of tutors thought that the cCBL sessions facilitated group discussions and thought them more interactive than the CBL’10 sessions; all had had experience with both formats.

‘Students were more engaged; the sessions generally were more interactive using breakout groups and post-it notes’. (Tutor 28).

‘Breakout stimulated discussion; smaller groups worked better, they were more interactive, more engaging and as a result, more enjoyable’. (Tutor 9).

‘Mini-group work is effective for discussion, the sessions are interactive, got you thinking about specific ideas first’. (Student 39).

As the small groups are presented with a set of clinical symptoms and a patient history in real-life cases, the discussions tend to draw on a wide range of topics and knowledge within the group.

‘Encourages discussion about dermatology + other systems’. (Student 86).

The biggest benefit—according to tutors and students—was the inclusivity, as the collaborative CBLs encouraged those who normally participate passively to engage better, due to the very small group size.

‘Definitely encourages more discussion and even from those who may not normally contribute’ (Tutor 16).

‘Allows to encourage even fairly non-responsive ones’ (Student 126).

With an increased engagement came a deeper understanding of the subject matter, and the students approached these sessions more confidently. Tutors commented that the students took control of their own learning and really practiced their clinical reasoning skills as intended, rather than relying on the tutors.

‘Encouraged students to identify own learning needs; onus is on students to breakout and discuss’. (Tutor 36).

‘Allowed students to be actively involved and develop clinical reasoning skills’. (Tutor 35).

‘Makes you think for yourself, got you thinking about specific ideas first and think of alternatives and different causes/potentials’. (Student 60).

2. Knowledge gain

Students felt that the interactivity helped contextualize the knowledge and they learnt better as a result of it; it was easier for them to concentrate on the topic when discussing it with their peers:

‘I felt like I learned more because I was more engaged as it was interactive; use of post-its was helpful in solidifying points from discussion and whether or not they were valid, in a way that previous CBLs lacked’. (Student 27, Student 20).

‘Very interactive which encouraged more active learning and discussions, were clinically relevant’. (Student 17).

Students felt that breaking down the topic to basics and building a hypothesis that they discussed with peers in smaller groups, allowed them to consolidate lectures.

‘Reinforces knowledge from lectures in a practical case situation; we learn more when we discuss the topic with others and it helps consolidate knowledge’. (Students 99 and 49).

‘[collaborative] CBLs wrapped up the lectures well and helped me to consolidate my knowledge’. (Student 42).

‘Breaking everything down to basics helped with understanding’. (Student 123).

‘Using ‘post-it's’ for differentials helps consolidate knowledge from the week; groups of 3 work best—groups should be really small, otherwise it doesn’t work’. (Student 21).

It should be noted, however, that a quarter of the students surveyed and responding were either neutral (17%) or disliked (8%) the discussions in breakout groups. These students questioned the usefulness of the approach as they felt they had insufficient knowledge of the subject matter to engage effectively in small group discussions.

‘The format was not very helpful; I prefer when it goes through the case sequentially; don’t like too many breakout group discussions – often we don’t know enough to comment’. (Student 84).

‘Students didn’t enjoy the post-it notes and preferred to discuss things as opposed to writing down’. (Tutor 8).

‘It is difficult to answer /discuss very broad topics in small groups with limited knowledge. It is much easier in the previous weeks to follow a case from start to finish with smaller questions’. (Student 91).

### Effect of the Introduction of cCBL on Summative Examinations

In order to objectively assess knowledge gain, we compared relevant exam performance of the cohorts that were taught specific topics via CBL’10 with those in later cohorts that received them as cCBLs.

**Fig. 2:**
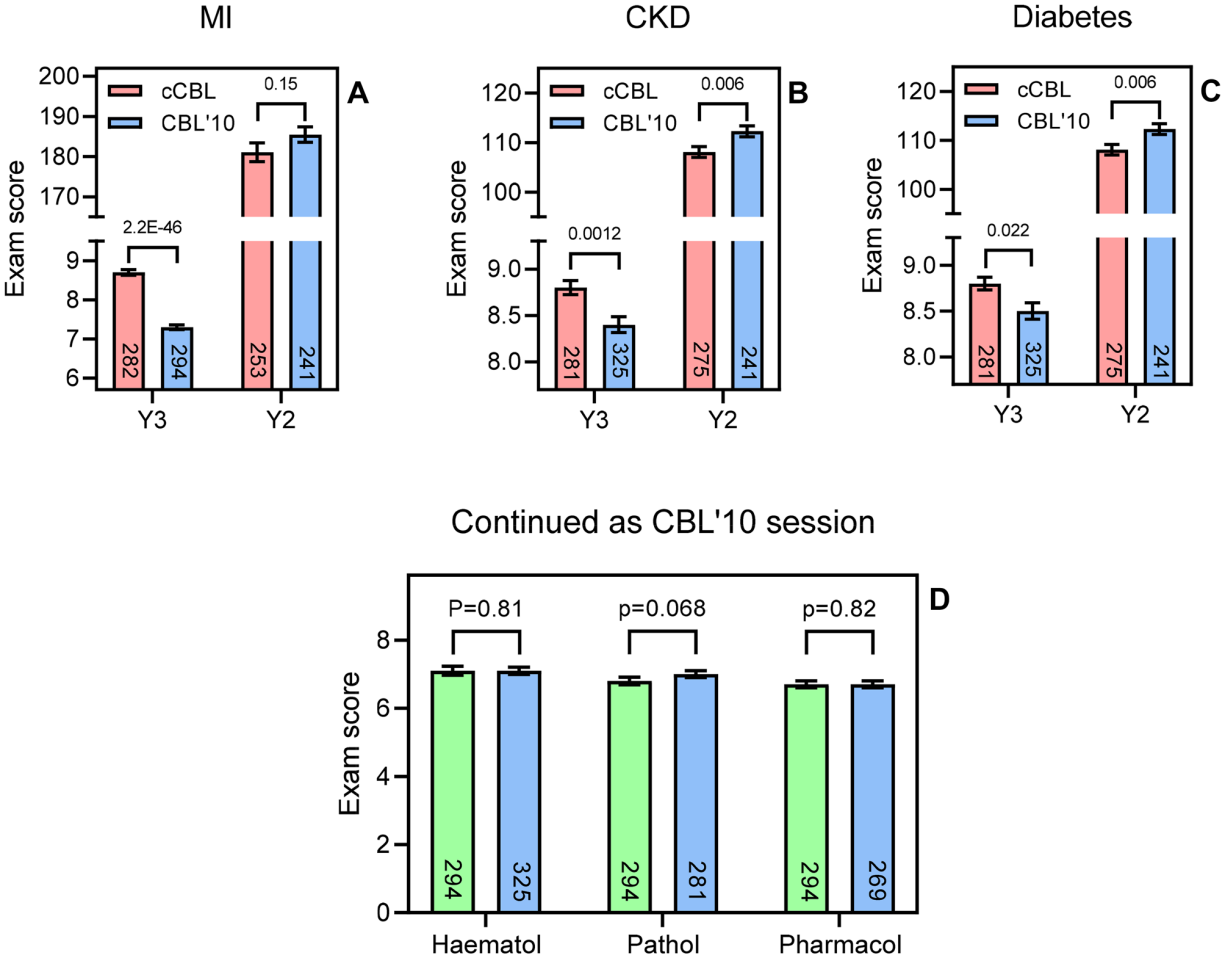
**A**–**C** Comparison of exam performance in year 3 on a specific topic between cohorts having been taught the subject by cCBL (red bars; 2019/2020 or 2020/2021) and CBL’10 (blue bars; 2015/2016 and 2018/2019). The two cohorts were set questions of comparable difficulty and complexity in their Y3 exams. As a control, the overall cohort performance in the end of year 2 exam was also analysed for the same cohorts. Bars show average ± SEM and the *t test* score is indicated above the compared bars. **D** Y3 exam performance comparison for the two cohorts in three subjects taught only via CBL’10 and assessed by a similar question on the topic. Green bars, cohort of 2019/2020; blue bars, cohorts of 2020/2021, 2018/2019, and 2015/2016, respectively. The number of students in each cohort is shown in each bar

There was a highly significant improvement in the cCBL students’ exam marks for the questions particularly on myocardial infarction (MI; *p* = *2.2E-46*, Fig. 2A), but also on chronic kidney disease (CKD; *p* = *0.0012*, Fig. 2B) and diabetes (*p* = *0.022*; Fig. 2C). As a control, the overall exam performance for the selected two cohorts at the end of year 2 was used, providing the nearest possible comparison. The two cohorts compared for MI, sitting the Y2 exam in 2018/19 (the cohort of Y3 in 2019–2020) and 2014/2015 (the Y3 cohort of 2015–2016), respectively, performed identically in this assessment, indicating that the two cohorts achieved comparable exam grades prior to cCBL (*p* = *0.15*; Fig. 2A). Analysis of the end-of-Y2 exam for the cohorts compared for CKD and diabetes showed that the cohort that would go on to receive CBL’10 in these subjects performed slightly better in the Y2 exam than the cohort that would go on to do cCBL in Year 3 (*p* = *0.006*; Fig. 2B, C).

However, not all topics in the 15-week Phase 3 were changed to cCBL at the same time. In order to allow a true side-by-side comparison, we analysed exam performance in topics that were still taught via CBL’10 in 2019/2020 (Fig. 2D, green bars) and compared these with exam performance in the same topic in previous years, also taught by CBL’10 (Fig. 2D, blue bars). In contrast to the gains seen in the cCBL topics of MI, CKD and diabetes seen in Figs. 2A–C, for the topics of haematology, pathology and pharmacology, taught via CBL’10 (and assessed in both cohorts), exam performance was statistically identical (*p* = *0.81*, *0.068* and *0.82*, respectively, Fig. 2D). We can thus conclude that the improvements in exam scores in cCBL-taught topics is likely attributable to the CBL format as no such improvement was seen when the same cohorts were compared in topics still taught via CBL’10.

## Discussion

CBL, in its original form [3, 31, 32], was introduced into our year 3 MBChB curriculum in 2010 and has consistently been one of the most highly evaluated components of the course. However, our tutor feedback indicated diminishing student engagement in the discussion elements of the class. Following the study by Krupat et al. [13], demonstrating an improved learning experience of students with the cCBL format, we introduced a similar change in academic year 2019/2020, in a subset of our CBL sessions, as a pilot study to address and investigate the student participation issues.

We have conducted a side-by-side comparison of the two cohorts that received both types of CBL and found that cCBL improved student engagement, motivation and knowledge gain and had a positive impact on assessment performance. Direct quantitative evidence of improved exam scores in the cCBL group was presented. Our findings are consistent with other studies that evaluated a switch to collaborative CBL [13, 33, 34], but our study is the first to compare two types of CBL in a mixed teaching year featuring both formats. While this study cannot firmly stipulate that the gain in the quality of learning by students can only be attributed to the switch from the original CBL to cCBL, no performance gain was seen in the subjects that remained taught as CBL’10. The majority of our students engaged well with the cCBL process and evaluated it positively.

We think cCBL sessions are particularly effective in developing clinical reasoning. The hypothesis-generating step and the discussions are opportunities for students to consolidate topics, first in small peer groups and then with the tutor for clarification, as required. This approach develops deeper clinical insights than didactic teaching can provide. The complex combination of skills and knowledge needed to arrive at differential diagnoses and evidence-based practice cannot be taught effectively via lectures alone [35, 36]. Interactive teaching sessions with peers and tutors, involving discussions and clinical reasoning, are essential and highly valued by the current generation of students [37]. The qualitative analysis carried out in this study identified two overarching themes: gain of engagement/motivation and gain of knowledge.

Fredricks et al. [38] distinguish three types of engagement: behavioural, emotional and cognitive, describing participation, emotional responses to the learning environment and deliberate investment of effort by the student, respectively. Expanding on this theme, studies by Wang and Eccles [39] and Kahu [40] as well as DiBenedetto and Bembenutty [41] found that each engagement type influenced academic performance and aspiration in different ways, but that both aspects were positively correlated with self-regulated learning. A study with 5,805 undergraduate students refined the model further to show that the effects of a student’s emotional state on achievement are mediated through self-regulated learning and motivation [42]. Cavanagh et al. [43] identified student buy-in of the active learning format as an important factor for improved course performance, showing the need to engage with the student body in co-creating and developing teaching material and the format in which the students want to be taught.

The data collected from students that participated in the cCBL sessions indicated that it enhanced learning, increased interactivity and promoted better learning of the topic through the group discussions. A large majority of the students believed that they learned more because of the interactive format and that the stepwise process of the cCBL enabled topics to be broken down, making the construction of hypotheses easier. They also reported that the collaborative format allowed better integration of diverse learning on the course, which built confidence and helped with consolidation. However, some of the students felt uneasy about being expected to discuss complex scenarios in such small groups, apparently not confident that they had the knowledge to do so. Table 1 shows that 12/149 responded disagree or strongly disagree to the survey question ‘The breakout sessions were useful in discussing topics with my peers’, with a further 25/149 responding ‘neutral’. It could be argued that these students may have actually benefitted most from this clinical reasoning training, as they will next find themselves in various ward placements requiring these skills.

The tutors found that the collaborative format made it easier to engage with, and motivate students, particularly the quieter ones, who had less opportunity to ‘hide’ in the small groups. While this may have been somewhat uncomfortable for the most passive participants, we regard the increased (need for) participation as one of the most positive outcomes of the cCBL format. Students are more anxious about being called upon (by the tutor) to answer a question in a larger group, as opposed to discussions with a few of their peers. McConnell and Eva [44] discuss how a (pre)-clinical student’s emotional state can determine learning outcomes and how direct questioning can induce ‘fear and stress’. The use of ‘post-it’ notes to capture ideas and thoughts is another effective way to get everyone involved in the process and can help overcome silence in groups as it serves to almost anonymize the opinions submitted to the larger group. Tutors also noted that cCBL helped students identify their own learning needs as the small group discussions readily identified knowledge gaps.

Almost all of the tutors (94%) surveyed felt more confident in leading the student discussions and that the session achieved the learning outcomes of the case. Thistlethwaite et al*.* [3] noted a similar satisfaction with CBL in tutors, attributing the enjoyment to either the use of authentic clinical cases, or the group learning effect. The cCBL development further extends these gains as our tutors noted that the smaller groups used in cCBL were probably the reason for the increased engagement. Moreover, tutors were more positive about delivering sessions because cCBL is more structured than CBL’10, which is hostage to the participation by students in the full group, with the iterative 3-step process providing tutors with a clear framework to follow.

The use of active learning is increasingly considered to be associated with student engagement and improved outcomes [45]. Bonwell and Eison [46] defined active learning ‘as instructional activities involving students in doing things and thinking about what they are doing’. It strengthens students’ use of higher order thinking to complete activities or participate in discussion in class, and it often includes working in groups. Students retain information for longer, as groups tend to learn through discussions, formulating hypotheses and evaluation of others’ ideas. Often, it helps them recognize the value of their contribution, resulting in increased confidence, as shown by the survey results. When views of cCBL were negative, it was almost always reflecting lack of confidence in their own knowledge, emphasizing the need to have CBL follow on from didactic teaching sessions, consolidating comprehension. This study confirms that cCBL encourages participation and that it is popular with most students, who find it a relevant way to prepare for the clinical phase of their medical education. Many students are still too passive in CBL’10, and the switch to cCBL aimed to increase ‘active learning’ for the entire cohort and thereby improve exam performance as well.

Although many medical schools are using active learning strategies, there is still little evidence in the literature that directly demonstrates a positive effect on summative assessment. Krupat et al. [13] showed an increase in attainment in lower aptitude students. In this study, we have shown exam performance improvements in all three subjects that switched to cCBL with a highly significant improvement in MI and modest improvements in two other topics. We attribute the very large improvement in cardiovascular question performance, relative to the more modest improvements in diabetes and nephrology, to the fact that the students have little cardiology teaching in year 2. In contrast, year 2 provided a solid understanding of the basic principles and clinical application of the other two subjects, which resulted in a less dramatic improvement when using cCBL, while the cardiovascular topic is a more sensitive test of the extent that knowledge gain is possible using cCBL. These increases in performance were compared to three other topics that remained taught by CBL’10 (haematology, pathology and pharmacology). None of these subjects showed any increase in assessment scores in the cohorts examined. Moreover, the cohorts performed virtually identically in their respective second year exams, suggesting that the difference in exam performance between the two cohorts taught via two different CBL formats could indeed be attributed to cCBL efficacy.

However, a limitation of any form of teaching is that one size rarely fits all. The collaborative style of CBL aims to encourage participation and discussion, with ample opportunity to explore issues in a safe environment, but we would wish to study how different individual learners perceive this and whether a more personalized approach could be developed. In addition, we would like to investigate whether non-specialty and specialty tutors find the process equally effective. The true benefit of the cCBL would have been logical to assess in a clinical setting, where the reasoning skills are exercised daily. However, it is obvious that there are a number of potential confounders to such a study evaluating students’ abilities to reason clinically, once they have more experience of placements.

To summarize, modification of CBL to a more collaborative approach with very small breakout groups is effective in improving medical students’ engagement with tutors and peers and their performance in assessment. This side-by-side direct comparative study outlines the clear benefits of the collaborative format: practicing clinical reasoning in small groups, and the power of the directed, focused discussions of the cases presented to the group led to increased participation as well as improved summative examination scores.
